# Induction of Allergic Responses to Peanut Allergen in Sheep

**DOI:** 10.1371/journal.pone.0051386

**Published:** 2012-12-19

**Authors:** Jenna L. Van Gramberg, Michael J. de Veer, Robyn E. O'Hehir, Els N. T. Meeusen, Robert J. Bischof

**Affiliations:** 1 Biotechnology Research Laboratories, Department of Physiology, Monash University, Clayton, Australia; 2 Department of Allergy, Immunology and Respiratory Medicine, Alfred Hospital and Monash University, Melbourne, Australia; University Hospital Freiburg, Germany

## Abstract

Peanut allergy is the leading cause of deaths due to food-induced anaphylaxis but despite continued research, there are currently no specific treatments available. Challenge testing is limited in patients due to the high risk of adverse reactions, emphasising the need for an appropriate animal model. In the present study we examine the induction of allergic responses in a sheep model for peanut allergy. Sheep were sensitised with peanut (PN) extract and in separate injections with ovalbumin (OVA) or house dust mite (HDM) extract. Serum PN-specific IgE responses were detected in 40–50% of immunised sheep, while only 10% (1 of 10 sheep) showed detectable OVA-specific IgE. All PN-allergic sheep tested showed an Ara h 1-specific IgE response, while four out of five allergic sheep showed an Ara h 2-specific IgE response. Animals with high serum IgE levels to HDM were also PN IgE-positive. Of the PN-sensitised animals with high PN-specific IgE, 80% also showed an immediate hypersensitivity reaction following an intradermal PN injection. This new large animal model of peanut allergy may provide a useful tool for future investigations of allergen-associated immune mechanisms and specific immunotherapy.

## Introduction

‘Food allergy’ can be described as an exaggerated immune response to certain ingredients present in food and chiefly characterised by the production of immunoglobulin E (IgE) antibodies [1], [2]. These IgE antibodies have been shown to have high affinity to receptors on immune cells such as mast cells and basophils [3] and interactions with these cells can lead to the release of potent mediators and the development of allergy symptoms [4], [5]. The symptoms of allergy can range from mild reactions (e.g. swelling, wheezing, vomiting/diarrhoea) to life threatening systemic anaphylaxis [6]. Adverse reactions to food account for 30–50% of emergency anaphylactic cases in North America, Europe, Asia and Australia, however, these rates are said to be even higher in paediatric patients [7].

The establishment of food allergies has been found to be more prevalent in the first two years of life. A 2011 study by Osborne *et al* [8], found that 10% of a sample of Australian infants had IgE-mediated food allergy, a rate they consider as being “higher than expected”. Food allergy sufferers also frequently experience other atopic conditions such as asthma, dermatitis (eczema) and rhinitis [9], [10]. This trend is consistent in peanut (*Arachis hypogaea*) allergy, with approximately 1% of children affected. However, unlike hypersensitivities to milk and egg, peanut allergies typically persist with age, becoming a lifelong burden on health and management [11].

Since peanut allergy is so prevalent and peanuts are the leading cause of deaths due to food-induced anaphylaxis globally [12], [13], considerable effort has been made to identify the major peanut allergens. As many as eleven peanut proteins have been categorised (Ara h 1–Ara h 11) [2] and, of these, Ara h 1 [14] and Ara h 2 [15] have been classified as major allergens, as they are recognised by serum IgE from more than 90% of peanut allergic subjects [16]. Further studies have reported that Ara h 2 is functionally more potent than Ara h 1 [17], [18] when comparing specific IgE and the ability of the protein to induce histamine release from basophils in sensitised subjects [19].

The use of animal models for the study of food allergens has continued to gain interest, since many key mechanisms and the observed responses mimic processes commonly seen in human allergic disease states [20]–[23]. Peanut allergy has been investigated in mice [21], [23], [24] as well as larger animals including dogs [22], [25] and pigs [20]. While animal models have furthered our understanding of the phenomenon that is allergy, larger animals are thought to reflect the human allergic state more closely due to their large size and outbred nature.

We have previously established a sheep model of allergic asthma based on house dust mite (HDM), that represents many features of the human disease including allergen-specific IgE responses, local and systemic inflammation and remodelling of the airways [26], [27]. The aim of the present study was to examine the induction of allergic responses to crude peanut allergens in sheep and establish the potential for this model in future studies of peanut allergy predisposition.

## Materials and Methods

### Experimental sheep and allergen sensitisations

All experimental animal procedures and the collection of tissues and cells were approved by the Animal Experimental Ethics Committee of Monash University, following guidelines set by the National Health and Medical Research Council (NH&MRC) of Australia. Merino cross ewes were treated with anthelminthic to eliminate any parasites prior, then housed in indoor pens and fed lucerne chaff for the duration of the experiment.

### Allergens

Crude peanut (PN) extract was prepared from commercial unsalted, dry-roasted peanuts as described [28], dialysed against phosphate-buffered saline (PBS) and filter-sterilised. Ara h 1 and Ara h 2 were each separated from crude peanut extract based on published methodology [29], [30] and kindly provided by Dr. Sara Prickett (Department of Immunology, Monash University). Ovalbumin (OVA) was purchased from Sigma. House dust mite extract (HDM; *Dermatophagoides pteronyssinus*; CSL Limited, Parkville, Australia) was prepared as previously described [26].

### Experimental Protocol

In Study 1 (n = 10) sheep were sensitised separately with a crude PN extract and OVA. The immunisation protocol involved 3 subcutaneous (s.c.) injections at 2-week intervals and a 4^th^ ‘boost’ injection after a rest period of 4 weeks. Each injection comprised either 100 µg of solubilised crude PN extract or 100 µg of OVA prepared in a total of 1 ml sterile saline with 50 µl of a commercial aluminium adjuvant (alum); Rehydragel ® LV- Aluminium hydroxide (Reheis Inc/NJ, USA). This protocol was derived from previous studies involving animal models that were immunised s.c. with allergen in alum [22], [26]. The PN injection was administered s.c. in the foreleg, whilst OVA was injected s.c. into the hindleg.

In Study 2 (n = 10), sheep were simultaneously immunised with PN allergen (as for Study 1) and HDM (50 µg/injection) allergen [26], prepared in 1 ml sterile saline with aluminium hydroxide as adjuvant. The immunisation protocol was similar to Study 1, with the PN and HDM injections administered s.c. at two different sites.

### Measuring antibody responses

Blood samples were collected from the jugular vein prior to the first immunisation (this served as a control for each individual sheep), as well as 4, 7, 14 and 21 days following the last injection. Serum samples were stored at −20°C prior to being assayed.

For the detection of PN-, HDM- or OVA-specfic total Ig by enzyme-linked immunosorbent assay (ELISA), ELISA plates were coated with 2.5 µg of the selected antigen in PBS, then washed, blocked and incubated with serum (1/200) for 1 hr at 37°C. After washing, plates were incubated with polyclonal rabbit anti-sheep horse-radish peroxidase (HRP; Dako, CA, USA) for 1 hr at 37°C, then washed and developed with 3′, 3′, 5′, 5′- tetramethyl-benzidine dihydrochloride hydrate (TMB; Invitrogen, VIC, Australia). Plates were read at 450 nm on a Molecular Devices SPECTRAmax PLUS spectrophotometer. All samples were tested in duplicate.

PN-, HDM- or OVA-specific IgE was determined by ELISA of NH_4_SO_4_-treated serum samples as described previously [26]. Briefly, equal volumes of serum and 60% NH_4_SO_4_ solution were mixed and the sample centrifuged after 30 min in a microcentrifuge (13000 rpm for 10 min). The supernatants (diluted 1∶1 with 0.1% Tween 20/dH_2_0) were added to 2.5 µg (PN/HDM/OVA) or 1 µg (Ara h 1/h 2) antigen-coated plates and incubated overnight at 4°C, followed by washing and incubation with anti-IgE mAb (clone XB6-YD3) [31]. Plates were then washed and incubated with HRP-conjugated (γ-chain specific) goat anti-mouse IgG (Sigma, NSW, Australia), then washed and developed with TMB-substrate. Plates were read at 450 nm on a Molecular Devices SPECTRAmax PLUS spectrophotometer. All samples were tested in duplicate.

### Skin testing

Immediate hypersensitivity responses were assessed following intra-dermal injections of 100 µl (1 µg/ml in saline) of PN, HDM and OVA; saline alone served as a negative control and histamine (1 µg/ml in saline) as a positive control. Injections were administered on the inner thigh of the animal. Thirty minutes after injection, wheal size was calculated as a mean of two measurements recorded using digital callipers [32].

## Results

### Serum Ig responses to PN extract and ovalbumin immunisations

**Figure 1:**
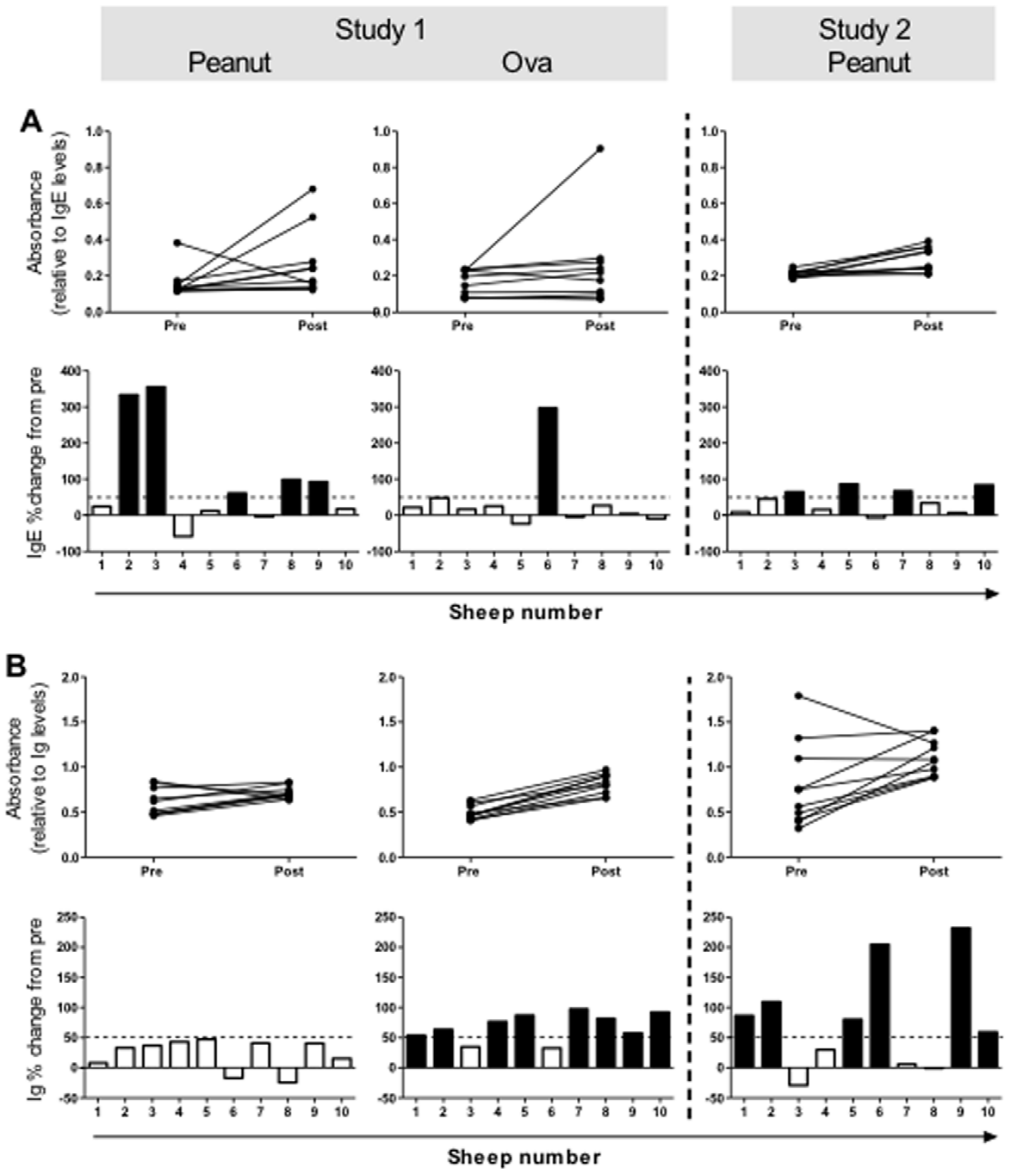
Serum Ig responses to peanut (PN) and ovalbumin (OVA) immunisations. Peripheral blood serum samples were collected at baseline (pre) and 7 days after the final PN/OVA immunisation (post). Line graphs depict allergen-specific IgE (A) and total Ig (B) levels (absorbance read at 450 nm) for individual sheep. Percentage change from baseline for each animal is presented in the bar graphs for allergen-specific IgE (A) and total Ig (B) levels. Solid bars illustrate a percentage change greater than 50%.

In Study 1, sheep were sensitised s.c. with crude PN extract and at a separate site with OVA, a commonly used model antigen. ELISAs were performed to determine PN/OVA-specific total and IgE levels in serum samples collected before (pre) and 7 days after the final injection. Sera collected prior to sensitisation served as a control for each animal. Sheep were classified as sensitised (allergic) on the basis of a 50% increase in PN-specific serum IgE levels. From the 10 sheep immunised, 5 animals developed a high PN-specific IgE response and were referred to as allergic sheep (Figure 1A, solid bars). Only 1 of the 10 immunised sheep developed a specific-IgE response to OVA (Figure 1A, sheep 6); this sheep was also allergic to PN. OVA, however, showed a greater percentage change for total specific-Ig levels in post-sensitisation sera, with 8 out of the 10 animals displaying an OVA-specific total Ig percentage change greater than 50% (Figure 1B). No change greater than 50% was seen in PN-specific total Ig responses (Figure 1B).

**Figure 2:**
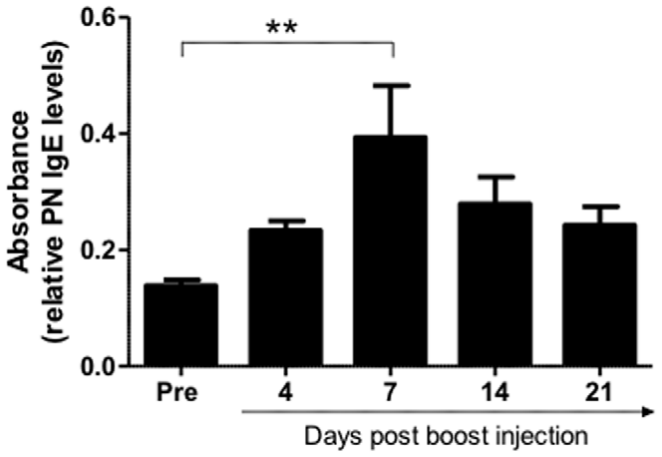
PN-specific IgE responses overtime in allergic sheep. Blood serum samples were collected before sensitisation (pre) and on days 4, 7, 14 and 21 after the final (boost) injection of PN extract, in order to track the peanut specific IgE antibody response overtime. Values are shown as the mean +/− SEM, n = 5 (data was analysed using a one-way ANOVA on repeated measures, Gaussian approximation); ** indicates a significant difference (p<0.01) comparing samples collected before and after PN exposure.

In order to track the specific IgE antibody response in the PN responders more closely, blood serum was also collected at days 4, 7, 14 and 21 after the final (boost) injection of PN extract. PN-specific IgE levels began to increase by the earliest time-point examined (day 4), with levels peaking at day 7 post boost and declining thereafter; these levels, however, remained higher than baseline levels as shown in Figure 2. A significant difference was seen in IgE levels between pre and day 7 sera levels (p<0.01).

In order to test reproducibility, an additional 10 sheep were immunised for Study 2. Sheep in study 2 were simultaneously sensitised with crude PN extract and HDM (s.c. injections in a separate site) to compare antibody responses to different allergens. A similar trend to Study 1 was seen with 40% of the sensitised sheep developing a PN-specific IgE response (Figure 1A), with no significant difference in the level of sensitisation to PN allergen comparing animals in Study 1 vs 2. Interestingly, while PN-specific IgE levels were similar to Study 1, the relative change in PN-specific total Ig levels appeared greater in Study 2 (Figure 1B). Sheep in Study 2 were also immunised with HDM allergen; 9 of 10 sheep became sensitised to HDM, and two sheep that showed the highest HDM-specific IgE levels were also positive for PN-specific IgE (data not shown).

### Serum IgE responses to the major peanut allergens Ara h 1 and Ara h 2

**Figure 3:**
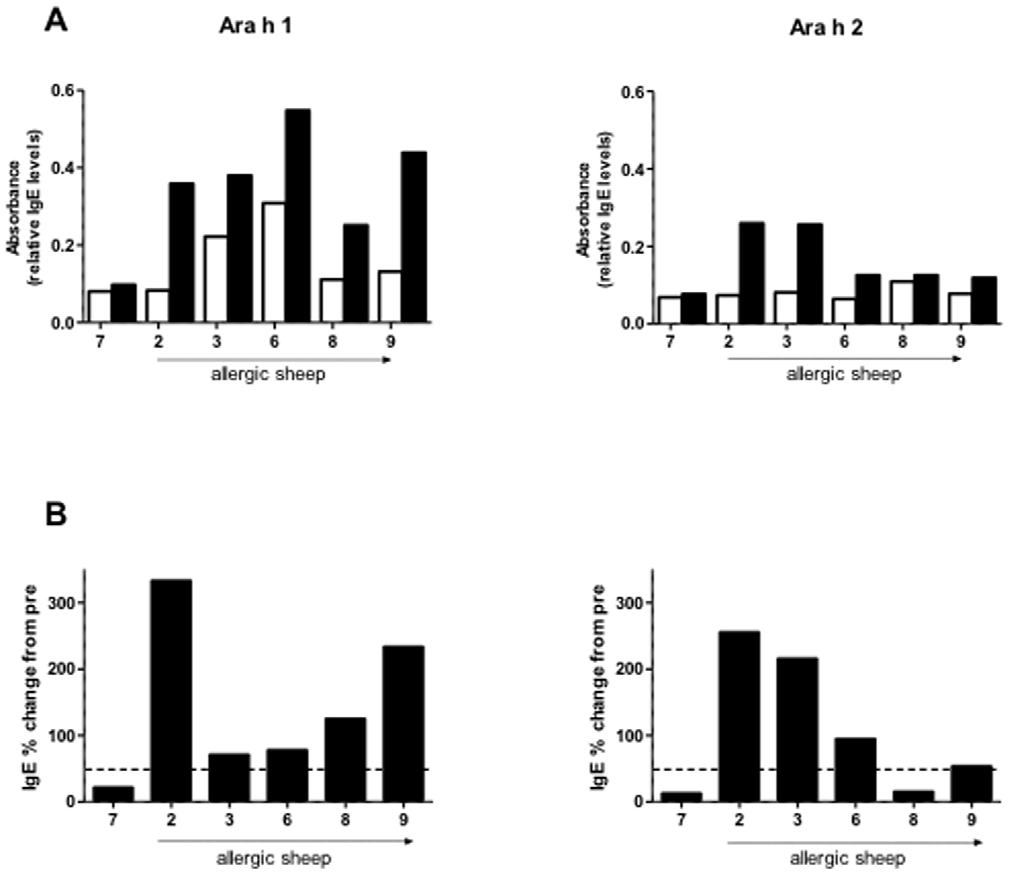
PN allergic sheep respond to the major peanut allergens Ara h 1 and Ara h 2. Blood serum collected before sensitisation (pre) and 7 days after the final (boost) injection of PN from allergic sheep (numbers 2, 3, 6, 8 and 9) and one non-responder (number 7) were tested for the presence of Ara h 1- and Ara h 2-specific IgE by ELISA. (A) Illustrates the individual differences between pre IgE (open bars) and day 7 post boost IgE levels (solid bars) whilst (B) shows the IgE percentage change from pre. Dashed line delineates greater than 50% change.

To detect whether the IgE antibodies raised against the PN extract were specific to the major PN allergens Ara h 1 and Ara h 2, further allergen-specific ELISAs were conducted. For PN-allergic sheep (n = 5) and one non-responder sheep (sheep 7), pre and 7 day post serum samples were tested for the presence of Ara h 1- and Ara h 2-specific IgE. All 5 PN-allergic sheep showed an Ara h 1-specific IgE response, while 4 out of 5 allergic sheep showed an Ara h 2-specific IgE response. The non-responder control (sheep 7) did not show an increased percentage change for either Ara h 1 or Ara h 2 (Figure 3).

### Skin wheal responses to crude PN and ovalbumin

**Figure 4:**
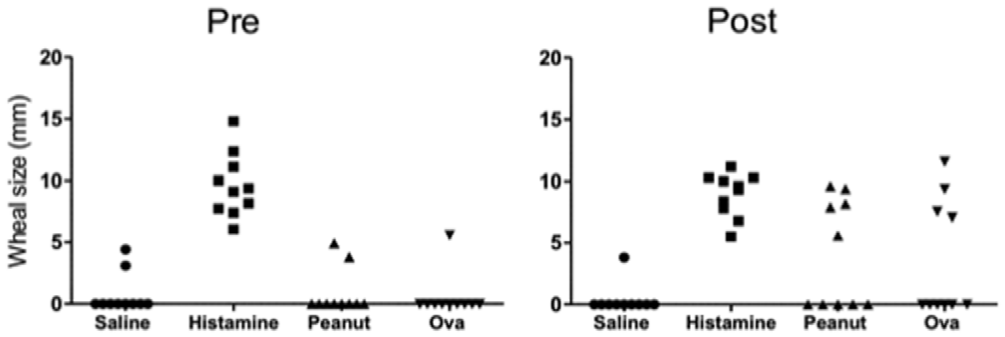
Skin wheal responses before and after allergen sensitisation. Mean wheal reaction size measured at 30 minutes post injection in Study 1 (n = 10). Saline alone served as a negative and histamine as a positive control.

Skin reactions following intradermal injections of PN extract and OVA were assessed in sheep of study 1 before (pre) and after (post) sensitisation. Saline served as a negative and histamine as a positive control. Immediate skin wheal reactions were observed and recorded at 30 minutes post injection in all sheep (Figure 4). Several sheep showed an unexpected wheal response to each of the injections at the pre-sensitisation stage. Three out of four sheep that showed a wheal response to OVA post sensitisation were also strong PN IgE responders. Four out of five animals that showed a strong IgE response to PN allergen (see Figure 1A) also gave a positive wheal response to PN post sensitisation.

## Discussion

In the present study we report the induction of allergic responses to peanut (PN) allergen in sheep, whereby sensitisation with a crude extract of PN induced high levels of PN-specific IgE in 40–50% of animals. Significantly, PN-allergic sheep showed strong IgE reactivity to two of the major peanut allergens, Ara h 1 and Ara h 2, which feature prominently in PN allergy in humans [14]–[16]. In addition to the systemic induction of PN-specific IgE, 80% of PN-allergic animals (4 of 5 sheep in Study 1) also displayed post-sensitisation wheal responses to PN allergen.

In a second study conducted here (Study 2), sheep were simultaneously sensitised with crude PN extract and a non-food allergen, house dust mite (HDM). The number of animals that displayed an elevated PN-specific IgE response (ie atopic for PN) was similar to that seen in Study 1, however, a more robust total Ig (presumably IgG1) response to PN allergen was observed in sheep in Study 2; most likely this was due to different genetic backgrounds of the sheep in each study and reflective of the outbred nature of this species (similar to humans). Sensitisation to HDM allergen is known to be a major contributing factor towards the development of allergic asthma in humans. In previous studies we used sheep as a model to characterise humoral and cellular immune responses after sensitisation with HDM extract and reported sensitisation rates of 50–60% [32], a rate similar to that seen in the present study using PN as the allergen. Comparing the sensitisation responses to PN and HDM allergen in the present study, we found that strong HDM-specific IgE responders were also positive for PN-specific IgE. This is in agreement with human studies, where it has been found that patients with asthma show an increased likelihood of sensitivity to other allergens including food allergens [33]. In individuals who experience anaphylaxis, a large proportion also display an asthmatic phenotype [7], [12], [34], [35] indicating a strong association (genetic predisposition) between these conditions.

The effects of sensitisation with PN allergen were also compared to ovalbumin (OVA), a common antigen used in murine allergy studies. In stark contrast to PN allergen, it was shown that OVA had a poor capacity for specific IgE induction (10% of immunised animals); this finding is supported in our own subsequent studies with 7 out of 76 animals showing Ova-specific IgE after sensitisation (Bischof *et al*, unpublished data). The discordance in the allergic response to PN compared with OVA may be due to inherent structural/protein differences between PN and OVA since it is well documented that peanuts have strong allergenic properties compared to other proteins. PN-induced allergic reactions are more severe compared to other food allergies [36] and are also a principal cause of IgE-mediated food allergy in humans, resolving infrequently and remaining a major source of morbidity in approximately 80% of allergic patients [2]. Interestingly, the present study showed that OVA immunisations result in elevated total OVA-specific Ig levels (80% of animals), despite the poor induction of OVA-specific IgE. Treatment success in immunotherapy trials is accompanied by increased IgG levels [37], suggesting that allergen-specific IgG may be able to block IgE-mediated reactions. Support for this was found in a murine model that showed antigen-specific IgG can block IgE mediated reactions through direct competition and signalling via the inhibitory IgG receptor FcγRIIb [38]. We showed in the present study that sheep can be sensitised concurrently with several food allergens (PN and OVA) and display differing levels of sensitivity/reactivity to these allergens, similar to previous studies in a dog model of food allergy [22].

In the present study we report that 2 of the 10 sheep tested showed a positive intradermal skin test to PN allergen at baseline (before PN sensitisation). False positive skin allergen tests have also been documented in other animal studies [20], as well as with human patients [4]. The existence of common carbohydrate cross-reactive determinants between PN-specific allergens and natural ‘environmental’ allergens may provide an explanation for the occurrence of false positive allergen sensitivity tests [39], [40]. Similarly, this may account for the revelation that 2 animals showed basal IgE reactivity to the peanut allergen Ara h1. In the clinical setting, intradermal skin tests for food allergy at times display poor specificity; positive skin reactions routinely require confirmation using other methods including blood testing or, where appropriate, food challenge [4].

A distinct advantage of large animals is their outbred status, which translates into a similar diversity of responses as seen in humans. The sheep model of PN allergy outlined here relies on sensitisation through subcutaneous injections of allergen in adjuvant (aluminium hydroxide), a protocol used widely in a range of animal models of allergy to induce significant levels of sensitisation and allow relevant studies on disease progression and responses to allergen provocation. While sensitisation to food allergens such as peanut is thought to principally occur via the oral route, other modes of sensitisation are likely, including respiratory and dermal routes [41].

Animal models have served to increase our understanding of the mechanisms responsible for allergy and have the potential to facilitate the development of safer therapeutic approaches. This study describes a new large animal model of peanut allergy that shows systemic IgE-responsiveness to peanut allergen and provides a robust system for investigative studies on allergen-associated immune mechanisms.
